# Pathogenesis of Molar Hypomineralisation: Hypomineralised 6-Year Molars Contain Traces of Fetal Serum Albumin

**DOI:** 10.3389/fphys.2020.00619

**Published:** 2020-06-12

**Authors:** Rebecca Williams, Vidal A. Perez, Jonathan E. Mangum, Michael J. Hubbard

**Affiliations:** ^1^Department of Pharmacology & Therapeutics, The University of Melbourne, Melbourne, VIC, Australia; ^2^Melbourne Dental School, The University of Melbourne, Melbourne, VIC, Australia; ^3^Department of Pediatric Stomatology, University of Talca, Talca, Chile; ^4^Department of Paediatrics, The University of Melbourne, Melbourne, VIC, Australia; ^5^Faculty of Medicine, Dentistry and Health Sciences, The University of Melbourne, Melbourne, VIC, Australia

**Keywords:** global health, paediatric disorders, dental defects, dental caries, medical prevention, developmental biomarkers, alpha-fetoprotein, biomineralisation

## Abstract

Molar Hypomineralisation (MH) is gaining cross-sector attention as a global health problem, making deeper enquiry into its prevention a research priority. However, causation and pathogenesis of MH remain unclear despite 100 years of investigation into “chalky” dental enamel. Contradicting aetiological dogma involving disrupted enamel-forming cells (ameloblasts), our earlier biochemical analysis of chalky enamel opacities implicated extracellular serum albumin in enamel hypomineralisation. This study sought evidence that the albumin found in chalky enamel reflected causal events during enamel development rather than later association with pre-existing enamel porosity. Hypothesising that blood-derived albumin infiltrates immature enamel and directly blocks its hardening, we developed a “molecular timestamping” method that quantifies the adult and fetal isoforms of serum albumin ratiometrically. Applying this novel approach to 6-year molars, both isoforms of albumin were detectable in 6 of 8 chalky opacities examined (corresponding to 4 of 5 cases), indicating developmental acquisition during early infancy. Addressing protein survival, *in vitro* analysis showed that, like adult albumin, the fetal isoform (alpha-fetoprotein) bound hydroxyapatite avidly and was resistant to kallikrein-4, the pivotal protease involved in enamel hardening. These results shift primary attention from ameloblast injury and indicate instead that an extracellular mechanism involving localised exposure of immature enamel to serum albumin constitutes the crux of MH pathogenesis. Together, our pathomechanistic findings plus the biomarker approach for onset timing open a new direction for aetiological investigations into the medical prevention of MH.

## Introduction

Molar Hypomineralisation (MH) is gaining cross-sector attention as a global health problem, making deeper enquiry into its prevention a research priority (Hubbard et al., 2017; Hubbard, 2018)^1^^,2^. Characterised by discoloured spots or patches of porous dental enamel (“demarcated opacities”) in one or more molars, MH puts children at risk of toothache and unusually rapid decay, often leading to costly management needs (e.g., ongoing restorations, extractions, orthodontics). MH affects the 2-year molars and/or 6-year molars of 1-in-5 children, together imposing massive social and economic burdens worldwide^3^^,4^. Invitingly, MH appears open to medical (primary) prevention, being developmentally acquired rather than primarily genetic in origin (Hurme, 1949; Suckling, 1989; Mangum et al., 2010; Hubbard et al., 2017).

Causation and pathogenesis of MH remain unclear despite 100 years of research into “chalky enamel,” leaving little hypothetical foundation for medical prevention. However, recent biochemical findings have led to a tentative aetiological breakthrough that challenges past thinking (Gottlieb, 1920; Suckling et al., 1976; Mangum et al., 2010). Longheld dogma maintains that demarcated opacities result from systemic disturbance of enamel-forming cells (ameloblasts) during the hardening (maturation) stage of enamel formation (Suckling, 1989; Suga, 1989; Jalevik et al., 2001; Weerheijm, 2003; Alaluusua, 2010; Fagrell et al., 2013; Babajko et al., 2017). Yet, despite bolstering general links to childhood illness, numerous epidemiological and laboratory studies have failed to identify a specific cause or pathomechanism (Alaluusua, 2010; Silva et al., 2016; Fatturi et al., 2019; Silva et al., 2019). In 2010, two proteomic investigations showed that chalky enamel opacities contained unusually high amounts of protein, including serum albumin and other derivatives of blood and saliva (Farah et al., 2010; Mangum et al., 2010). Provocatively, our biochemical follow-up showed that the protein composition of chalky enamel varied drastically depending on integrity of the opacity surface. After finding albumin as the only abundant protein in intact opacities, it seemed that an extracellular (rather than cell-based) mechanism might disrupt mineralisation directly (Mangum et al., 2010). Importantly however, although a potential role had previously been recognised for albumin due to its mineral-binding properties (Robinson et al., 1992; Robinson et al., 1994), widespread skepticism about experimental artefact in animal models prevailed (Couwenhoven et al., 1989; Chen et al., 1995; Wright et al., 1997; Takagi et al., 1998; Mangum et al., 2010; Robinson, 2014). Aetiological significance was further clouded by contradictory findings concerning the normal existence of albumin in human enamel (Farah et al., 2010; Mangum et al., 2010).

The present study sought evidence that albumin found in chalky enamel opacities reflects causal events during enamel development and not later incidental associations. Hypothesising that albumin infiltrates immature enamel and directly disrupts its mineralisation, the initial goal was to distinguish this mechanistic possibility from later adsorption of albumin to pre-existing enamel porosity. We developed a unique “molecular timestamping” approach that quantified the adult and fetal isoforms of albumin ratiometrically, then applied it to chalky enamel isolated from 6-year (first permanent/adult) molars. Having supportively found traces of fetal albumin in intact opacities, attention turned to how it survived there during the proteolytic onslaught of enamel hardening.

## Materials and Methods

### Specimens, Biologicals, and Biochemicals

All specimens from human subjects were obtained with informed consent under institutional ethical approval (HEC 0719683, The University of Melbourne). Extracted 6-year molars were stored unfixed at −80°C essentially as before (Mangum et al., 2010) except that prior washing was done with physiological saline instead of water. Neonatal serum was collected from healthy newborns in three samplings during the first postnatal week then pooled and stored at −80°C (gift from Prof. Paul Monagle, Department of Paediatrics, The University of Melbourne). Human albumin (Sigma), alpha-fetoprotein (Lee Biosolutions), recombinant alpha-fetoprotein and albumin (both tagged with glutathione S-transferase; from Abnova), and rabbit polyclonal antibodies to human albumin (PAB10220 from Abnova), alpha-fetoprotein peptide (PAB12795 from Abnova) and whole alpha-fetoprotein (H00000174-D01 from Abnova), were obtained commercially. Amelogenin extracts were prepared from developing rat molars as before (Mangum et al., 2010). Thermolysin and all other reagents were from Sigma (analytical grade), unless stated otherwise.

### Molecular Timestamping of Early Infancy

**FIGURE 1:**
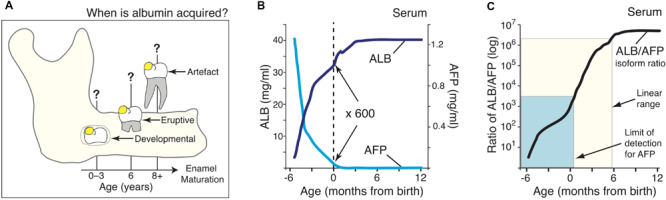
A molecular approach for timestamping exposures of serum albumin to dental enamel. **(A)** Schematic showing three hypothetical stages where albumin could become incorporated in enamel on a 6-year molar (*yellow spot*), as considered in this study. These albumin exposures are designated *developmental* (0–3 years), *eruptive* (6 years), or *artefactual* during subsequent extraction, as indicated. **(B)** Serum levels of serum albumin (ALB; *dark blue*) and alpha-fetoprotein (AFP; *cyan*) in healthy individuals aged from 3-months postconception through to 1-year old, as indicated. Data were collated and averaged from 9 population studies as outlined in Methods and Supplementary Figure S1. It can be seen that at birth (*dotted line*), ALB is about 600-fold more concentrated than AFP. **(C)** The adult/fetal isoform ratio for serum albumin (*black line*), as derived from **(B)**, rises quasi-linearly through to 6-months of age (*cream box*). Sensitivity limits for the AFP immunoblot assay (Figure 2) reached the early-post-natal period (*cyan box*). Note the isoform ratio, which spans 6-orders of magnitude (cf. log scale), offers strong resolution of age in early infancy.

A blood-based molecular timestamp for the perinatal period and early infancy was developed bioinformatically. First, population-average data were derived for serum levels of the fetal and adult isoforms of albumin (alpha-fetoprotein and serum albumin, respectively). Literature values at various ages were collated from 9 studies of healthy, full-term subjects as detailed in Supplementary Figure S1. Second, the average values for each isoform were ratioed to provide a normalised measure immune to individual variations in serum albumin concentration. Averaging and ratioing were done by non-linear curve-fitting (cubic spline fit) using Prism software (GraphPad). Quasi-linearity of the ensuing albumin/alpha-fetoprotein ratio (Figure 1C) enabled its use as a molecular timestamp for calibrating the onset of chalky enamel development, as outlined in the text.

### Profiling of Enamel Proteins

Paediatric dentists (RW, VP) diagnosed MH using standard criteria for demarcated opacities (Suckling, 1998; Weerheijm et al., 2003). Chalky demarcated opacities bearing a visibly intact (shiny) surface were selected for analysis, and those with surface breakdown (cracking, chipping, pitting or caries involvement) were excluded to avoid contamination by oral fluid proteins (Mangum et al., 2010). This study employed a total of 15 opacities taken from 6 molars (i.e., representing 6 MH cases). Chalky enamel, defined as discoloured (cream/yellow/brown) enamel removable with hand tools (Mangum et al., 2010), was harvested with a scalpel and/or slowly rotating bur (No. 2 tungsten carbide from Komet) and the collected powder measured volumetrically using a calibrated 1 μl micro-spoon (Fine Science Tools). Enamel-protein samples taken from chalky and control (normal) enamel were acid-precipitated then solubilised at room temperature in reducing SDS-PAGE sample buffer containing protease inhibitors as before (Mangum et al., 2010). Equivalent enamel volumes were analyzed by SDS-PAGE using precast mini-gels (AnyKDa mini-protean TGX, from BioRad, with Tris/glycine buffer) followed by Coomassie Blue staining. Protein size (*M*_r_, expressed as kDa for brevity) was calibrated with a prestained ladder (Precision Plus Dual Colour Protein standards, from BioRad), and average nominal values for serum albumin (65 kDa), alpha-fetoprotein (70 kDa) and enamel albumin (70 kDa) were derived by semi-log plot. Note these values differ from classical determinations made with unstained protein ladders (Hubbard, 1995; Mangum et al., 2010) and also from later experiments done with different batches of (commercial) gels. Immunoblotting was done using optimised electrotransfer conditions (wet tank method), probing (overnight incubation in primary antibody, rapid handling thereafter), and colorimetric detection (Vectastain ABC alkaline phosphatase kit, from Vector Labs) as previously (Mangum et al., 2010; Perez et al., 2018). Standard antibody dilutions were: anti-albumin 1:2,000; anti-alpha-fetoprotein peptide, 1:500; anti-(whole alpha-fetoprotein), 1:200. Where indicated, avidin/biotin-blocking was performed in Tris-buffered saline using streptavidin (0.1 mg/ml for 15 min) then biotin (0.5 mg/ml for 60 min) before the primary-antibody step. Sample loadings were adjusted to give detection within the linear range established by imaging densitometry of serially diluted standards (Perez et al., 2018) except where indicated. Spiking with tagged recombinant proteins (albumin, alpha-fetoprotein) that migrated slower than native protein standards was used to establish detection sensitivity for complex specimens containing native albumin/alpha-fetoprotein (i.e., neonatal serum, opacities).

### Proteolysis Assay

Albumin, alpha-fetoprotein, or amelogenin substrates were incubated at 37°C with matrix metalloproteinase-20 (MMP20; recombinant human catalytic domain, from Enzo Life Science) or kallikrein-4 (KLK4; recombinant human pro-KLK4, from R&D Systems) under standard conditions (Li et al., 1999; Ryu et al., 2002; Tye et al., 2009; Perez et al., 2018). Briefly, MMP20 (60 or 180 ng) was incubated with 1 μg substrate in 10 μl of buffer (50 mM Tris-HCl pH 7.5, 10 mM CaCl_2_, 150 mM NaCl, 50 μM ZnCl_2_) for times indicated in the figure legends. Pro-KLK4 was activated by incubation with thermolysin, which was subsequently inactivated with 1,10-phenanthroline according to the supplier’s instructions. Activated KLK4 (60 or 180 ng) was incubated with 1 μg substrate in 10 μl of buffer (50 mM Tris-HCl pH 7.2, 5 mM CaCl_2_).

### Other Methods

Mineral-binding assays were done by incubating proteins with powdered pure hydroxyapatite under limiting conditions as before (Mangum et al., 2010; Perez et al., 2018). Automated assay of alpha-fetoprotein in extracts of chalky enamel was attempted using a standard clinical immunoassay (AVIA Centaur, with AFP ReadyPack Lite reagent; from Siemens) with appropriate controls for the protein extraction procedure. Digital image manipulation was limited to linear brightness and contrast adjustments at whole gel/blot level, and selected areas were composited as described in the figure legends. Original images of whole gels/blots were provided for review.

## Results

### Ratio of Adult to Fetal Serum Albumin Is a Molecular Timestamp for Perinatal Development

Three opportunities for exposure of enamel to serum albumin were considered in 6-year molars: tooth development inside a baby’s jaw; tooth eruption near 6 years; and later tooth extraction (Figure 1A). Seeking to delineate the first period, neonatal blood was noted to be distinguished by the residual presence of alpha-fetoprotein (AFP), the fetal isoform of serum albumin (Bader et al., 2004). Reasoning that AFP might become incorporated in chalky enamel if exposure occurred during infancy, normal blood concentrations of AFP and (adult) serum albumin (ALB) were collated from 9 studies and then averaged by curve-fitting (Figure 1B; see also Supplementary Figure S1). Given the ALB content of chalky opacities was quite variable (Mangum et al., 2010), we implemented a ratiometric approach to compensate for individual differences. The resulting ratio between adult and fetal albumins (hereafter termed the “isoform ratio”) exhibited a quasi-linear relationship with age up to 6-months old, and involved a 400-fold increase between birth and 3 months (Figure 1C; *cream box*). We concluded that the isoform ratio of albumin provides a “molecular timestamp” for the perinatal period that might elucidate the pathogenesis of MH.

### High-Sensitivity Quantification of the Isoform Ratio for Serum Albumin

**FIGURE 2:**
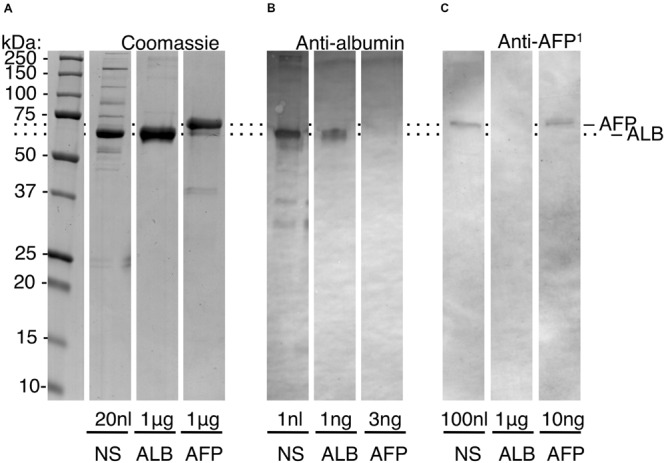
High-sensitivity quantification of the adult/fetal isoform ratio for serum albumin. Protein standards were subjected to SDS-PAGE and immunoblotting (alkaline phosphatase detection) as described under section “Materials and Methods.” Sample loadings are given to enable semi-quantification. Relative mobilities of AFP and ALB were normalised (*dotted lines*) given minor variability across experiments. Within-experiment resolution of AFP from ALB is illustrated in Supplementary Figure S3. **(A)** Coomassie Blue-stained gel, showing neonatal serum (NS), and pure standards (ALB, AFP) as indicated. **(B)** Albumin-immunoblot, showing non-detection of AFP at 3-fold excess over ALB. **(C)** AFP-immunoblot developed with peptide antibody (anti-AFP^1^), showing non-detection of ALB at 100-fold excess over AFP. Comparison of **(B,C)** reveals that AFP is a minor constituent of neonatal serum, as expected from Figure 1. This figure is composited from **(A)** a single Coomassie-stained gel, and **(B,C)** single immunoblots for each antibody.

In healthy full-term newborns, blood (serum) levels of AFP are ∼600-fold lower than ALB, necessitating high-sensitivity assays (Figures 1B,C). Initial experiments showed that a standard medical assay for AFP (using a monoclonal antibody) was incompatible with the harsh conditions used for isolating enamel proteins (not shown). Accordingly, we turned to SDS-PAGE and immunoblots realising this approach could beneficially disclose protein degradation, as previously seen with ALB (Mangum et al., 2010). Using antibodies against a peptide unique to AFP, a high-sensitivity assay was established (detection limit <5 ng AFP) and found to totally discriminate AFP from ALB, even at 1,000-fold excess of the latter (Figure 2, and data not shown). When applied to neonatal serum, an isoform ratio of about 500 was obtained in accordance with literature values (Figure 1B). We concluded that the immunoblot assay was valid for quantifying isoform ratios of albumin from prenatal through to early-postnatal ages (Figure 1C; *cyan box*).

### Trace Detection of Fetal Albumin in Hypomineralised 6-Year Molars

**FIGURE 3:**
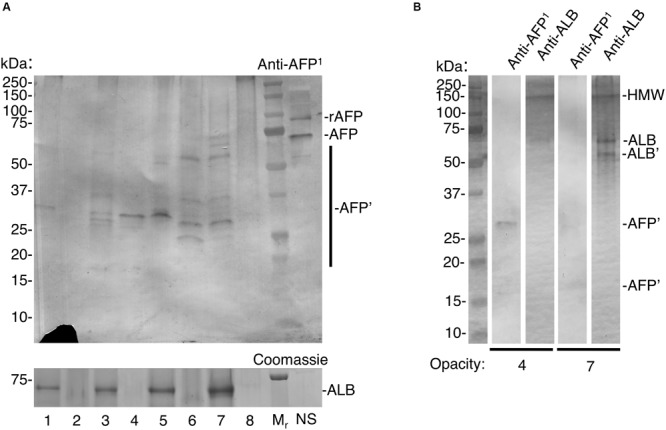
Trace detection of fetal albumin in hypomineralised 6-year molars. Chalky opacities and neonatal serum (NS) were subjected to SDS-PAGE and immunoblotting as in Figure 2, with the addition of a biotin-blocking step as described under Methods. Intact opacities were taken from a single 6-year molar representing each of 5 cases as follows: case 1, opacity 1; case 2, opacities 2,3,4; case 3, opacity 5; case 4, opacities 6,7; case 5, opacity 8. In **(A)**, AFP detection sensitivity was established using incremental loadings of 93 kDa recombinant AFP (rAFP), run both spiked into NS (interference test; 3 ng) and alone (pure standard; with 3/10/30 ng) as illustrated in Supplementary Figure S3. **(A)** Equalised samples from opacities 1–8 were analysed alongside molecular weight markers (*M*_r_) and NS, using AFP-immunoblot and Coomassie Blue staining (for ALB) as indicated. Note that AFP-fragmentation patterns (AFP’) appear unrelated to ALB levels, consistent with specific immunodetection of AFP. The recombinant AFP marker was readily immunodetected at 3 ng loading both when spiked into NS (rAFP) and run alone (Supplementary Figure S3), indicating lack of interference under these conditions. As expected the 3 ng rAFP band was undetectable by Coomassie staining (30 ng was clearly detectable; not shown). The lack of an ALB band is because NS was omitted from this gel due to scarcity of supply. **(B)** Re-run immunoblot analysis of opacities 4 and 7 from **(A)**, probed in parallel for AFP and ALB as indicated. Note distinct band patterns were revealed with each antibody, indicating again that AFP-immunodetection was specific. For ALB, a distinctive fragment (ALB’) and high molecular weight aggregate (HMW) are marked. The diminution of some bands (compared to **A**) has been observed with re-run samples previously (Mangum et al., 2010). This figure is composited from **(A)** a single immunoblot and parallel Coomassie-stained gel, and **(B)** a single gel immunoblotted for AFP and ALB in parallel.

At face value, the above considerations implied that AFP would be detectable in chalky enamel from 6-year molars if exposure to AFP occurred neonatally and the infiltrating fluid had a similar isoform ratio as blood. Initial trials on intact opacities revealed specific labelling of AFP-like bands, but non-specific labelling of two known biotin-containing proteins (Hubbard, 1995) was also observed. Accordingly, a biotin-blocking step was added to erase the non-specific labelling. After biotin-blocking, AFP-specific bands were detected in low amounts by the AFP-peptide antibody (Figure 3A; see Supplementary Figure S2 for control blot). When compared against neonatal serum, AFP in chalky enamel specimens (hereafter “enamel AFP”) was seen to be variably degraded. Seven discrete fragments of AFP could be discerned across 2 or more opacities (see Supplementary Figure S3). High molecular weight bands (>250 kDa) were also present, consistent with the known propensity of AFP to aggregate and degrade (Wu and Knight, 1985). Verifying the absence of cross-reaction between protein standards (Figure 2), the relative amounts of AFP and ALB varied between opacities (Figure 3A) and parallel probing of individual opacities revealed distinct patterns for AFP and ALB (Figure 3B). The identification of AFP was further validated by reprobing the immunoblot with an antibody against whole AFP. Not only were some AFP bands enhanced, but distinctive new bands coinciding with ALB were also revealed, consistent with known cross-reactivity to ALB (Supplementary Figure S3). Overall, AFP fragments were detected convincingly in 6 of 8 opacities examined (corresponding to 4 of 5 MH cases). We concluded that AFP was present at trace levels in a majority of chalky opacities, and that its partial degradation and aggregation were consistent with longterm residency therein.

### Does Fetal Albumin Signal a Neonatal Onset for Hypomineralised 6-Year Molars?

**FIGURE 4:**
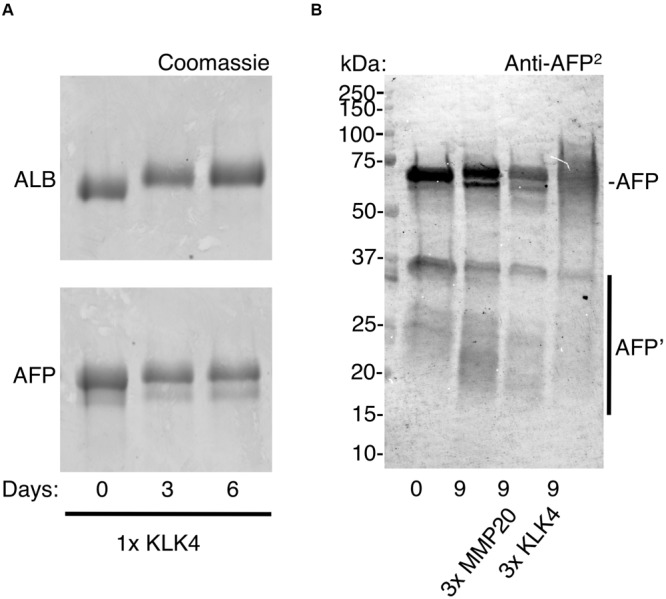
Resistance of ALB and AFP to enamel proteases. Protein standards (ALB, AFP) were exposed to enamel proteases (KLK4, MMP20) at 37°C, as described under Methods. After various times as indicated, digests were analysed by SDS-PAGE and immunoblotting as above except that an antibody to whole AFP (anti-AFP^2^) was used. **(A)** Coomassie staining reveals similarly high stability of ALB and AFP after 6 days in moderate proteolytic conditions (1× KLK4 = 60 ng). **(B)** Immunoblotting reveals that AFP was partially degraded after 9 days under harsher proteolytic conditions (3× MMP20/KLK4 = 180 ng), as reflected by depletion of the parent AFP band and near-absence of AFP fragments. Note that significant degradation also occurred in the absence of added protease, with fragments being more obvious (*lane 2*, bands at 15–30 kDa). Digest samples were deliberately overloaded to enhance visibility of AFP fragments and so the band pattern for intact AFP should be regarded as semi-quantitative only. This figure is composited from a single **(A)** Coomassie-stained gel, and **(B)** immunoblot.

Although degradation of adult and fetal albumins hindered their quantification, the isoform ratio in chalky opacities grossly matched that of neonatal serum (Figures 2, 3). So does this mean the exposure to serum albumins, and postulated onset of hypomineralisation, occurred neonatally? For the blood-based timestamp (Figure 1C) to apply accurately in developing enamel, it would be necessary for AFP and ALB to have equivalent stabilities against enamel proteases, and also for both isoforms to be retained equally through binding to enamel mineral. In the case of protein stability this appeared untrue given AFP was more highly fragmented than ALB (Figure 3A). Indeed, ALB is a robust protein with known resistance to kallikrein-4 (KLK4), the pivotal protease that degrades amelogenin – the predominant protein in developing enamel – and so enables enamel to harden (Takayama et al., 2001; Mangum et al., 2010). In contrast, AFP reportedly has intrinsically poor stability particularly when isolated from blood (Wu and Knight, 1985). Asking whether AFP is similarly resistant to KLK4 as ALB, protein standards were exposed to moderately high amounts of the protease for 6 days at 37°C and analysed by SDS-PAGE with Coomassie staining. Finding that KLK4 had little effect on both albumin isoforms whereas amelogenin was degraded completely as expected (Figure 4A, and data not shown), a longer timecourse was examined by immunoblotting (Figure 4B). After 9 days without added protease, AFP remained largely intact, yet an accumulation of 15–30 kDa fragments was revealed by immunoblotting (Figure 4B, *lane 2*). Intriguingly, these fragments loosely matched those predominating in opacities (Figure 3A, and Supplementary Figure S3). When KLK4 was added at threefold higher concentrations than before, the amount of intact AFP decreased substantially as did the 15–30 kDa fragments (Figure 4B, *lanes 3 and 4*). Another enamel protease, matrix metalloproteinase-20 (MMP20), also appeared partially active toward AFP under these harsh conditions (Figure 4B, *lane 3*; and data not shown). Together these findings suggest that KLK4 exhibits only modest activity toward AFP, and that ensuing fragments are unstable in its presence. Instead, the lingering AFP fragments observed in chalky enamel (Figures 3A, 4B) may reflect intrinsic instability of AFP (Wu and Knight, 1985). Addressing retention on enamel crystals, which may also be crucial to protein survival (Mangum et al., 2010; Perez et al., 2018), we established that AFP and ALB standards exhibited similarly avid binding to hydroxyapatite (not shown) as expected (Ido and Matsuno, 1982). Unfortunately this question could not be tackled for AFP fragments due to a lack of suitable digests. We concluded firstly that the trace amounts of AFP detected in hypomineralised 6-year molars (Figure 3A) are consistent with neonatal exposures and not with the older ages under consideration (Figure 1A). Secondly, accuracy of the isoform-ratio approach is hampered by stability differences of AFP and ALB in chalky enamel.

## Discussion

If MH is to be managed better and ultimately prevented, fuller understanding of its molecular pathology is essential. Contradicting aetiological dogma about systemic injury to ameloblasts, biochemical analysis of chalky enamel opacities has shifted attention to direct disruption of enamel hardening by serum albumin. Our results support this extracellular mechanism by revealing that both the fetal and adult isoforms of albumin are present in chalky enamel isolated from 6-year molars, consistent with developmental acquisition during early infancy and not later. Together, these pathomechanistic findings plus the new “molecular timestamp” approach for onset-timing open a new direction for aetiological investigations into the medical prevention of MH.

Drawing on well-established medical data, we have derived a powerful molecular approach for timestamping perinatal events involving serum albumin (Figure 1). The steep rise in isoform ratios provides a sensitive measure of age extending from 6 months before through to 6 months after birth (>6 orders of magnitude; Figure 1C, *cream box*). Medically, this whole period is measurable in blood samples due to the high sensitivity of automated assays for AFP. Hence, albumin-isoform ratiometry might prove clinically useful for profiling various pathophysiological changes, noting that AFP and ALB levels vary in prematurity and disease (Polberger et al., 1990; Blohm et al., 1998; Bader et al., 2004). Our necessitated shift to immunoblot analysis proved advantageous by revealing AFP fragmentation that otherwise may have escaped detection (Figure 3). However, due to its limited sensitivity, the immunoblot assay was ineffective beyond the early-postnatal period (Figure 1C, *cyan box*). It seems likely that, due to their earlier development, primary (baby) teeth will be more readily measured with the current assay and that further methodological development will enable a longer reach into infancy (Figure 1C).

Addressing our primary goal of distinguishing developmental exposures to albumin from later possibilities (Figure 1A), AFP clearly had potential to be an unambiguous discriminator for some cases of MH. For example, even by 1-year of age, blood levels of AFP are >6 orders of magnitude lower than ALB, hence falling far below our detection limit (Figures 1B,C). The identification of AFP fragments in intact opacities (Figure 3) appeared robust based on three biochemical criteria (i.e., specific labelling with peptide and whole-protein antibodies, fragments in common with serum AFP but not enamel ALB, varied albumin ratios across different opacities). Consequently, these results provide the first solid evidence of serum albumin (fetal and adult isoforms) having infiltrated and been retained in developing (chalky) enamel. This result accords with our other findings about surface impermeability of intact chalky opacities (Mangum et al., 2010). So, for 6-year molars at least, albumin is firmly implicated as a direct inhibitor (“poison”) of enamel mineralisation as suggested previously from circumstantial evidence (Robinson et al., 1992, 1994; Mangum et al., 2010; Robinson, 2014). A novel second element of our renovated “mineralisation poisoning” proposal is that mineral-bound albumin survives the proteolytic removal of amelogenins during enamel hardening, thereby providing a permanent arrest of crystal growth that leads to porous chalky enamel (Mangum et al., 2010; Perez et al., 2018). This aspect was supported by our findings that AFP exhibited ALB-like resistance to KLK4 (Figure 4) and affinity for hydroxyapatite, provisionally explaining the observed retention of AFP fragments within opacities (Figure 3). While longstanding concerns about artefactual associations of albumin with porous enamel remain valid (Couwenhoven et al., 1989; Chen et al., 1995; Wright et al., 1997; Takagi et al., 1998; Farah et al., 2010; Mangum et al., 2010; Robinson, 2014), such concern appears untenable for the AFP results reported here. Collectively therefore, our findings lead us to conclude that serum albumin plays a direct role in the pathogenesis of MH.

A successional goal, to use albumin-isoform ratiometry to timestamp the onset of MH, was partially successful and so further pursuit of this approach seems worthwhile. At face value, near-equivalence of the isoform ratios detected in chalky opacities and neonatal serum (Figure 3) suggests that, in those cases, the defective enamel had been exposed to serum albumin soon after birth. That multiple opacities had similar isoform ratios also suggests a surprisingly narrow window of risk, albeit a larger dataset will be required before drawing conclusions. Later onsets are implicit for those opacities in which AFP was undetected (2 of 8 examined), but the sensitivity limitation precludes assignments of onset age. However, while likely valid in gross terms, such timings must be approached with caution for several reasons. Foremost, the isoform ratiometry was calibrated with population-average data for intact albumins from blood of healthy, full-term subjects (Figure 1C). Noting that common neonatal conditions (e.g., prematurity, jaundice and hepatitis) trigger elevated AFP (Bader et al., 2004), it seems plausible that our MH subjects deviated from this normal baseline – in which case, MH-onset age would be underestimated (Figure 1C). Moreover, AFP in chalky enamel was fragmented (Figure 3), complicating quantification and inferences made about mineral binding. Additional complexity surrounds the high standard-deviation of normal AFP concentrations in blood (Bader et al., 2004) and potential differences from AFP levels in tissue fluid. Further efforts to address these challenges are warranted given the double attractions of gaining unprecedented spatiotemporal resolution (i.e., biomarker analysis of individual opacities) and identifying a narrower risk window for MH. Presently, aetiological investigations are hampered by poor understanding of the vulnerability period (reportedly extending from late prenatal to 3–4 years old for 6-year molars; Beentjes et al., 2002; Fagrell et al., 2013; Silva et al., 2016; Fatturi et al., 2019) during which myriad maternal and childhood illnesses prevail.

This study involved microscale biochemistry on scarce specimens and so inevitably has several limitations including the issues of sensitivity, calibration and quantification already noted. Moreover, having focussed on AFP and early pathogenesis (medical onset), questions remain about the predominance of ALB at later stages (dental outcomes). Of critical importance however, our biochemical approach has led to unequivocal identification of AFP in demarcated opacities for the first time.

In conclusion, this study breaks new ground by revealing trace amounts of AFP in chalky enamel and establishing a biomarker approach for timing the onset of hypomineralised 6-year molars.

These advances have narrowed initial pathogenesis to the early postnatal period and eliminated long-standing concerns about artefactual binding of ALB. Consequently, we believe this study and our related findings (Mangum et al., 2010; Perez et al., 2018) justify reconsideration of the dogma that systemic injury to ameloblasts is the pathological crux of MH. Instead, aetiological attention should turn to the pathomechanistic prospects and allied causality surrounding localised exposures of immature enamel matrix to serum albumin. Having weathered 100 years of mystery about causation and pathogenesis, it is hoped this new research direction will ultimately lead to medical prevention of MH and consequent benefits for global health.

## Data Availability Statement

All datasets generated for this study are included in the article/Supplementary Material.

## Ethics Statement

The studies involving human participants were reviewed and approved by the University of Melbourne human Ethics Committee. Written informed consent to participate in this study was provided by the participants’ legal guardian/next of kin.

## Author Contributions

MH and JM contributed to the project conception and design. RW, VP, JM, and MH contributed to the experimental design, data analysis, interpretation, and thesis chapters. MH, VP, JM, and RW contributed to the final manuscript, read and approved the final manuscript.

## Conflict of Interest

MH is the founder/director of The D3 Group for Developmental Dental Defects (thed3group.org, a charitable network). The remaining authors declare that the research was conducted in the absence of any commercial or financial relationships that could be construed as a potential conflict of interest.

## Footnotes

1 www.thed3group.org

2 www.chalkyteeth.org

3 www.thed3group.org/prevalence.html

4 www.thed3group.org/social-impact.html

## Abbreviations

AFP: alpha-fetoprotein
ALB: serum albumin (adult)
HMW: high molecular weight
KLK4: kallikrein-4
MH: molar hypomineralisation
MMP-20: matrix metalloproteinase-20
NS: neonatal serum.
