# Supplementary material for: Pathogenesis of Molar Hypomineralisation: Hypomineralised 6-Year Molars Contain Traces of Fetal Serum Albumin
Source: Front Physiol. 2020 Jun 12;11:619. doi: 10.3389/fphys.2020.00619 (PMC7303361; doi:10.3389/fphys.2020.00619)
Supplement: Supplementary file 1 [file Data_Sheet_1.pdf]

Pathogenesis of Molar Hypomineralisation: hypomineralised 6-year molars contain traces of fetal serum albumin (R Williams, VA Perez, JE Mangum, MJ Hubbard)

SUPPLEMENTARY FIGS.

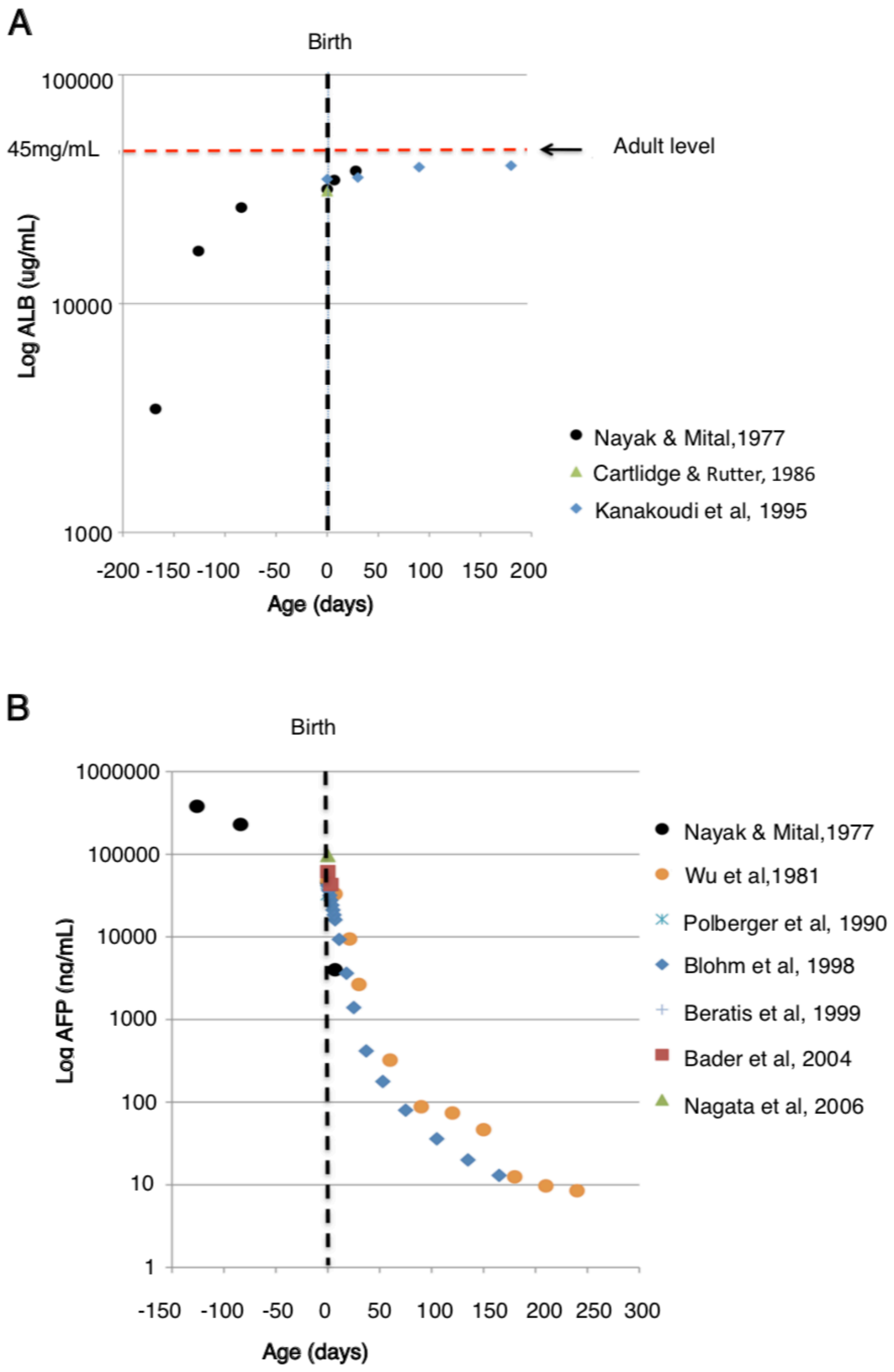

Supplementary Figure S1. Serum concentrations of ALB and AFP diverge in the perinatal period.
Serum concentrations of ALB and AFP at various (mean) ages were taken from 9 population studies and plotted on a semi-log scale as indicated. **(A)** Serum ALB levels rise rapidly during the prenatal period and then plateau at near-adult levels shortly after birth. **(B)** Serum AFP levels

remain similarly high prenatally then fall exponentially during the first 100 days after birth before plateauing at trace levels (≈ 10ng/ml) from 200 days on. Some cited articles can be found under References in the main text (Polberger et al., 1990; Blohm et al., 1998; Bader et al., 2004) and the others by using the following identifiers at the PubMed database (ncbi.nlm.nih.gov/pubmed): Nayak, 1977 (65131); Cartlidge, 1986 (3740904); Kanakoudi, 1995 (7536645); Wu, 1981 (6163129); Beratis, 1999 (10412820); Nagata, 2006 (16896034).

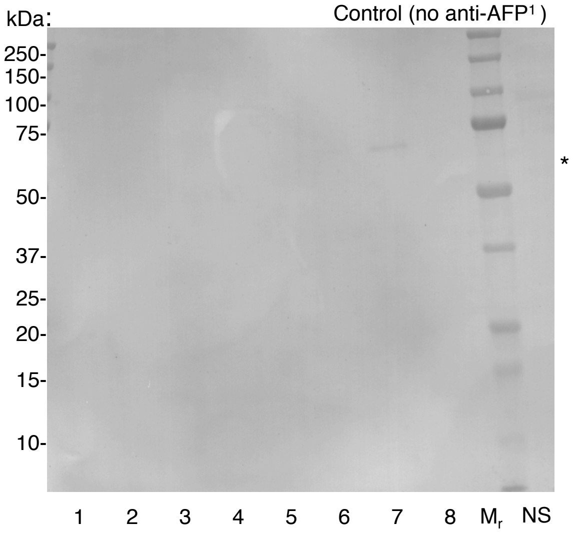

Supplementary Fig. S2. Control immunoblot for Figure 3A.
A partner blot to that shown in Figure 3A was probed exactly in parallel except that primary antibody (anti-AFP[1]) was omitted. In comparison with Fig. 3A where numerous AFP-positive bands were revealed across most specimens, such bands were absent from this control blot. A known biotinylated protein that hadn't been fully silenced by the biotin-blocking step (see text) was detected weakly in opacity 7 at about 60-kDa (*asterisk*).

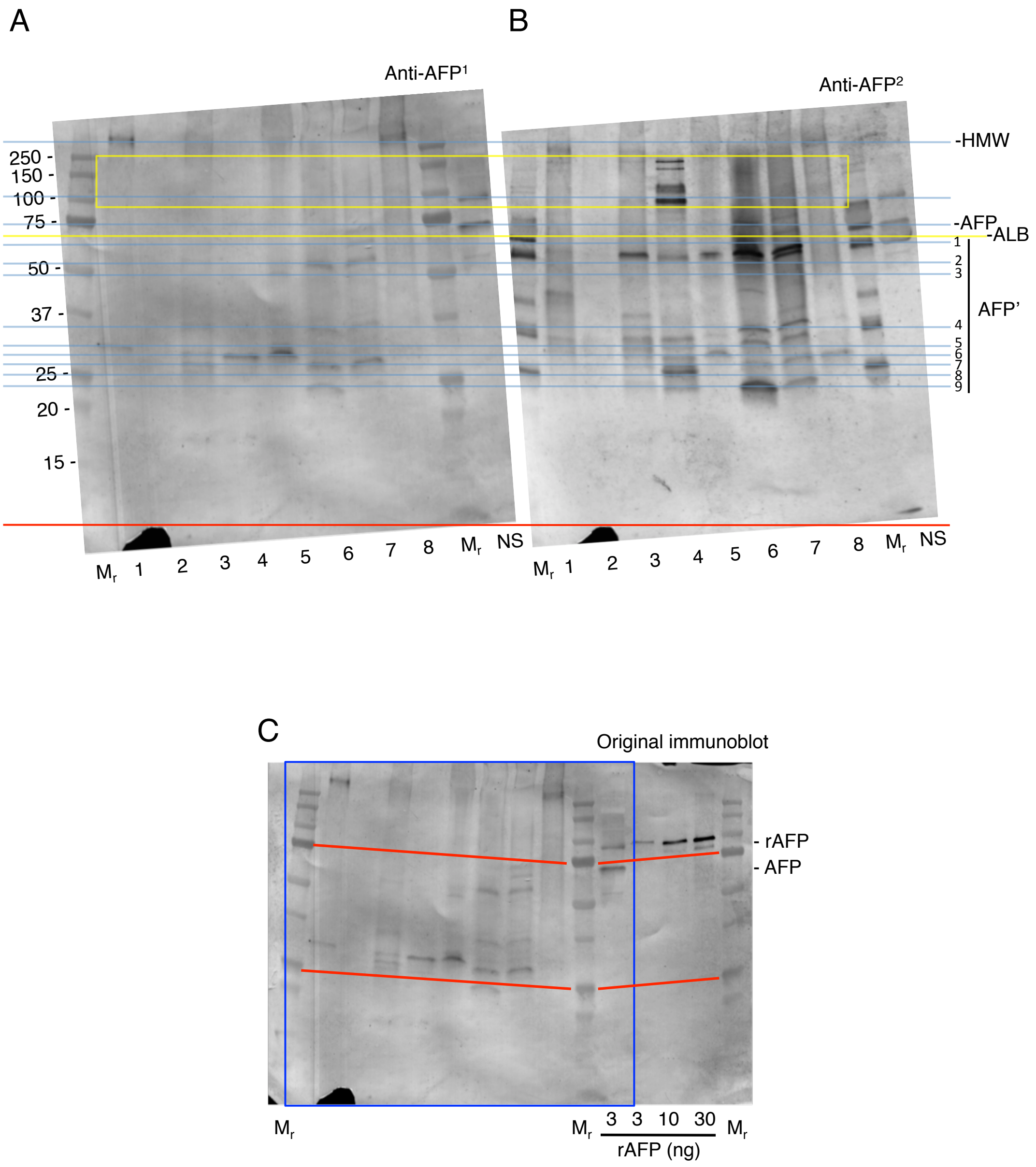

Supplementary Figure S3. Reprobe analysis of Figure 3A using a less-specific antibody
The blot previously developed with monospecific anti-AFP[1] (**A**, reproduced from Figure 3A) was reprobed with an AFP-antibody (anti-AFP[2]) that cross-reacts mildly with ALB (**B**). No membrane-stripping step was employed before reprobing. Comparing neonatal serum (NS) lanes, ALB is detected by anti-AFP[2] only (*yellow line*). Comparing opacities, anti-AFP[2] also strongly detects multiple bands undetected by anti-AFP[1], indicating these are ALB species (e.g. *yellow box* and *yellow line*). Conversely, the anti-AFP[1] pattern is not markedly altered by anti-AFP[2], in both the high and low molecular weight regions (*blue lines*). Nine apparent fragments of AFP, 7 of which recur in two or more specimens, are indicated (AFP'$_{1\text{-}9}$). Some of the low molecular weight bands (putative AFP fragments; AFP') are more prominent in **B**, consistent with anti-AFP[1] and anti-AFP[2] being peptide and whole-protein antibodies, respectively. Together with the control blot (Supplementary Fig. S2), it can be concluded that the bands in Figure 3A are correctly designated as AFP species. **(C)** Intact immunoblot marked up to show the foundation for rotating the images as in **A** and **B**. That this gel had a curved migration front ("smile") is evident from the red lines joining the M_r markers (75 kDa, 25 kDa). The cropped region is indicated (*blue box*). Loadings of recombinant AFP standard (rAFP), both spiked into the NS lane and alone, are indicated (3-30 ng).
